# Optimization of Protective Agents for The Freeze-Drying of *Paenibacillus polymyxa* Kp10 as a Potential Biofungicide

**DOI:** 10.3390/molecules25112618

**Published:** 2020-06-04

**Authors:** Hayatun Syamila Nasran, Hidayat Mohd Yusof, Murni Halim, Nor’Aini Abdul Rahman

**Affiliations:** 1 Department of Bioprocess Technology, Faculty of Biotechnology and Biomolecular Sciences, Universiti Putra Malaysia, Serdang, Selangor 43400, Malaysia; hayatun.nasran@gmail.com (H.S.N.); hidayatmy@gmail.com (H.M.Y.); murnihalim@upm.edu.my (M.H.); 2 Bioprocessing and Biomanufacturing Research Centre, Faculty of Biotechnology and Biomolecular Sciences, Universiti Putra Malaysia, Serdang, Selangor 43400, Malaysia

**Keywords:** antimicrobial, antifungal, biofungicide, optimization, *Paenibacillus polymyxa*, RSM

## Abstract

Anthracnose is a fungal disease causing major losses in crop production. Chemical fungicides widely used in crop plantations to combat fungal infections can be a threat to the environment and humans in the long term. Recently, biofungicides have gained much interest as an alternative to chemical fungicides due to their environmentally friendly nature. Biofungicide products in powder form can be formulated using the freeze-drying technique to provide convenient storage. Protective agent formulation is needed in maintaining the optimal viable cells of biofungicide products. In this study, 8.10 log colony-forming unit (CFU)/mL was the highest cell viability of *Paenibacillus polymyxa* Kp10 at 22 h during incubation. The effects of several selected protective agents on the viability of *P. polymyxa* Kp10 after freeze-drying were studied. Response surface methodology (RSM) was used for optimizing formulation for the protective agents. The combination of lactose (10% *w*/*v*), skim milk (20% *w*/*v*), and sucrose (27.5% *w*/*v*) was found to be suitable for preserving *P. polymyxa* Kp10 during freeze-drying. Further, *P. polymyxa* Kp10 demonstrated the ability to inhibit fungal pathogens, *Colletotrichum truncatum* and *C. gloeosporioides*, at 60.18% and 66.52% of inhibition of radial growth, respectively.

## 1. Introduction

Anthracnose is a common disease caused by fungal pathogens affecting crops’ yield and fruit quality [1]. *Colletotrichum capsici, C. gloeosporioides*, and *C. acutatum* have been reported as pathogens causing chili anthracnose [2]. Conventional fungicide is a chemical substance commonly used in crop plantations to combat fungal diseases. However, the unregulated use of chemical fungicides can be detrimental to public health especially to people that consume crops as a fiber source. Moreover, the excessive use of chemical fungicides can also contribute to environmental problems through the contamination of groundwater where fungicides are absorbed into the soil [3]. Therefore, an urgent strategic approach is needed to replace the presently used fungicides. Biocontrol agents, defined as the use of an organism to reduce the population of another organism, have now been introduced to replace the harmful chemical pesticides [3]. For successful commercializing of biocontrol agents, some product development strategies including preservation steps have to be implemented for maintaining high cell viability throughout the process.

*Paenibacillus* species has the ability to be applied as an alternative biocontrol agent to the current chemical pesticides against plant pathogens. The species produces substances that can be used as pesticides and utilized for bioremediation agents. For instance, *Paenibacillus polymyxa* strains possess potential antagonistic activity, where they can secrete antimicrobial compounds that demonstrate a spectrum of activity against fungi, bacteria, and nematodes [4,5]. Further, *P. polymyxa* strains were reported to produce a range of antibiotics such as polymyxin, polypeptins, and jolipeptin, which effectively work against bacteria, and also produced gatavalin and fusaricidin against fungi [6,7]. In addition, they offer protection against various insect herbivores and phytopathogens by triggering a hypersensitive defensive response of the plant, known as induced systemic resistance [8]. *P. polymyxa* strain (HY96-2) isolated from a tomato rhizosphere has proven to control tomato bacteria wilt and provide beneficial effects in promoting tomato plants’ growth [9]. Likewise, *P. polymyxa* NSY50 isolated from vinegar waste compost was identified as a potential biocontrol agent for controlling *Fusarium* wilt, a major destructive soil-borne disease infecting cucumber [10]. Furthermore, *Paenibacillus* also has the potential as a biocontrol for food-borne bacteria including *Salmonella* [11]. Putting the wide antagonistic activity features of *P. polymyxa* into consideration, this species could be the best candidate for biofungicide.

The success in commercializing bacterial strains as a potential biofungicide is highly dependent on preservation technologies employed by industrial companies. Suitable preservation methods could maintain high cell viability not only during the preservation process but most importantly during the long-term product storage [12]. Since bacteria have vulnerable characteristics, freeze-drying under vacuum or lyophilization is often considered as one of the proper preservation methods of the cells. The freeze-drying technique begins with the frozen step followed by primary drying through sublimation and desorption as the second drying stage [13,14].

Several factors affect the viability of cells during the freeze-drying process. In general, freeze-drying may cause two different stresses onto the cells. The first is where the cells are frozen and water molecules in the cells are disabled followed by a drying stage where water molecules are removed [15]. Ice formation in the cells during the freezing phase could cause damage to the cells due to membrane rupture [16]. To overcome these effects, protective agents are usually added before freezing or freeze-drying stage [17,18]. However, there are many other factors affecting bacteria survival such as intrinsic factors that include bacteria morphology [19], growth medium used during bacteria culture [20], bacteria survival during the culture environment [21,22], and storage conditions after preservation [23]. Thus, this present study aims to optimize the protective agents for *P. polymyxa* Kp10 subjected to freeze-drying using response surface methodology (RSM) to obtain the optimal number of cells after the freeze-drying process and to investigate the biofungicide potential of *P. polymyxa* Kp10 against fungal phytopathogen which commonly affects crop production.

## 2. Results and Discussion

### 2.1. Cell Growth Curve of P. polymyxa Kp10

**Figure 1:**
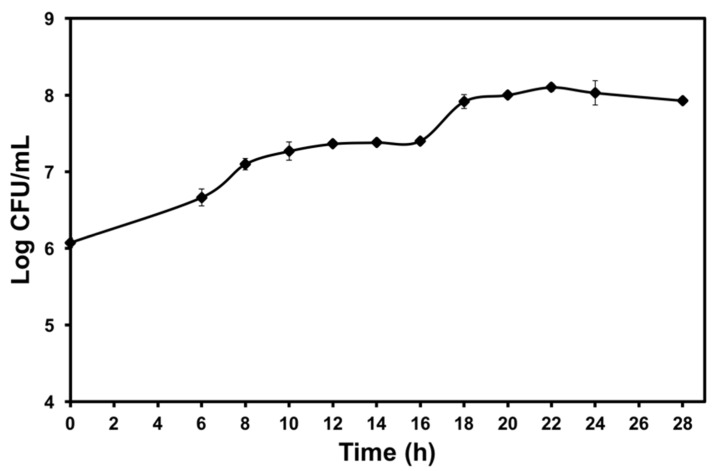
A growth profile of *P. polymyxa* Kp10 based on colony-forming unit (CFU)/mL (♦) during 28 h of the incubation period. The error bars represent the standard deviations about the mean (*n* = 3).

Figure 1 shows that the highest cell viability of *P. polymyxa* Kp10 was 8.10 log colony-forming unit (CFU)/mL attained at 22 h of the incubation period. The cell growth started to gradually decrease at 24 h with its cell viability at 8.03 log CFU/mL. According to Keivani et al. [24], bacteria are more resistant in the stationary phase since they develop general stress resistance due to nutrient exhaustion compared to bacteria conditions in the log phase. *P. polymyxa* GBR-1 also has its maximum cell viability between 20–28 h after incubation [25]. In general, the survival rate of cells depends largely on the type of organisms. In an earlier report, *Lactobacillus rhamnosus* at the stationary phase gave the highest recovery rate (31%–50%) after drying compared to the cells at the early log phase with 14% survival rate [26]. Likewise, *Rhizobia* cells from stationary phase achieved higher cell viability compared to those in the log phase [27]. Thus, in this particular work, 8.10 log CFU/mL of cells harvested at 22 h are considered as a suitable number for an initial cell concentration to be subjected to freeze-drying for minimizing the possible cell loss after the freeze-drying process.

### 2.2. Selection of Protective Agents for Bioformulation of P. polymyxa Kp10

Skim milk was shown as the most suitable protectant agent for *P. polymyxa* Kp10 cells in maintaining high cell viability compared to control and Soytone (Table 1). Skim milk (20% *w*/*v*) provided the highest protection for cell survival at 89.26% followed by lactose (10% *w*/*v*) with 87.78% survival rate. Skim milk is one of the most preferable protective agents and widely studied for freeze-drying of various types of bacteria [16,28,29,30]. Proteins and calcium in the skim milk may contribute to cell protection from the extreme and harsh conditions of the freeze-drying process by forming a protective coating on the cell wall [31]. Sucrose provides 83.14% survival rate of *P. polymyxa* Kp10 as the protective agent. Hubalek [32] suggested that sucrose at the concentration of 10% (*w*/*v*) is the most frequently used for microorganism lyophilization. Meanwhile, Ming et al. [16] used sucrose at 20% (*w*/*v*) for freeze-drying *L. salivarius* I 24 and obtained a considerably high cell survival rate of 9.0%.

As expected, distilled water that was used as a control for the protective agents’ selection for *P. polymyxa* Kp10 recorded a very low survival rate (62.24%) as there was no protection of the cells during lyophilization. A similar result was observed when Soytone (69.13% *w*/*v*) was used as the protective agent in which only a 0.46% survival rate was recorded after freeze-drying. Likewise, Portner et al. [33] reported that 10% (*w*/*v*) Soytone gave less optimal protection for *Campylobacter jejuni* as a protective agent during freeze-drying. Based on this study, skim milk, lactose, and sucrose were the preferred protective agents for *P. polymyxa* Kp10 and thus they were chosen for the subsequent optimization study.

### 2.3. Optimization of Protective Agent Combination Using RSM

Protective agent combinations were discovered based on a five-level two-variable central composite design as shown in Table 6, which comprised the actual factor level corresponding to coded factor levels. Regression analysis was used on experimental data and several prediction models are shown in Table 2.

ANOVA illustrated that the optimization of protective agent combination was most preferably described to the quadratic model as shown in Table 3. ANOVA shows a lack of fit that was not significant (*p* > 0.05), hence defining that the models are significant and can be used for optimization. The *F*-value for the model was 182.58, which was significant. AB, AC, BC, A^2^, B^2^, and C^2^ were the significant model terms. Model terms of A, B, and C were not significant since their *p*-value (Prob > *F*) were more than 0.05. Lack of fit was not significant with the value of 3.52. There was a 9.68% chance that lack of fit value could happen because of noise. In determining the fitness of the model, the model must have the significance of the model (*p* < 0.05) and the insignificance of the lack of fit (*p* > 0.05) [34]. Fitness between the development model and experimental data can be determined based on the coefficient value (R^2^). In this study, R^2^ was equal to 0.9940, implying low error in the model.

**Figure 2:**
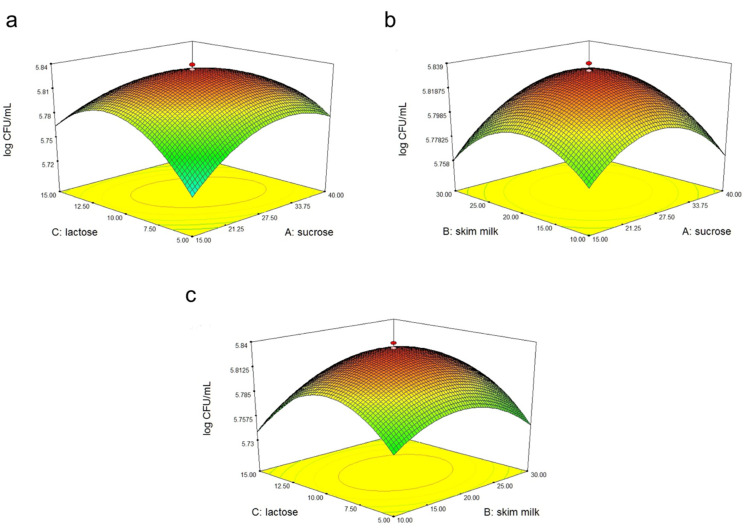
Response surface plots for cell viability of *P. polymyxa* Kp10 affected by (**a**) lactose (10% *w*/*v*) and sucrose (27.5% *w*/*v*); (**b**) skim milk (20% *w*/*v*) and sucrose (27.5% *w*/*v*); and (**c**) lactose (10% *w*/*v*) and skim milk (20% *w*/*v*) as protective agents subjected to freeze-drying.

Figure 2 shows the response surface curves plotted in function of two factors while the third was maintained constant at its main level. Based on the three graphs plotted in Figure 2, the maximum cell viability after freeze-drying was predicted at 5.839 log CFU/mL when lactose (10% *w*/*v*), sucrose (27.5% *w*/*v*), and skim milk (20% *w*/*v*) were used as the combined protective agents for *P. polymyxa* Kp10. This suggests that the combination of lactose and sucrose could give a synergetic effect in protecting the cells during the process. In addition, it can be seen that the combinations from the selected protective agents provided a synergic effect in maintaining high cell viability after the freeze-drying process. Sucrose was reported to replace water around polar residues in macromolecular, thus stabilizing cell membranes and proteins during desiccation [35]. Sugars from disaccharides could preserve cell structure by hydrogen bond formation that maintains tertiary protein structure when water molecules are absent [36]. Meanwhile, skim milk rich in protein is capable of preventing cells from injury caused by extracellular ice formation during the freezing stage by providing a protective coat to the cells [32]. The survival rate of cells after freeze-drying for lactose (10% *w*/*v*) and sucrose (27.5% *w*/*v*) was 83%. According to Wong et al. [37], 71.65%–82.07% of survival rates can be obtained when using different skim milk and sugar combinations as protectants. This supported the survival rate of *L. salivarius* at 83%–85% when a combination of skim milk, trehalose, and sucrose was used as a protective agent [37]. In this study, a combination of skim milk, sucrose, and lactose gave a significant effect on cell viability after the freeze-drying process by maintaining maximum cell viability.

### 2.4. Antifungal Activity

**Figure 3:**
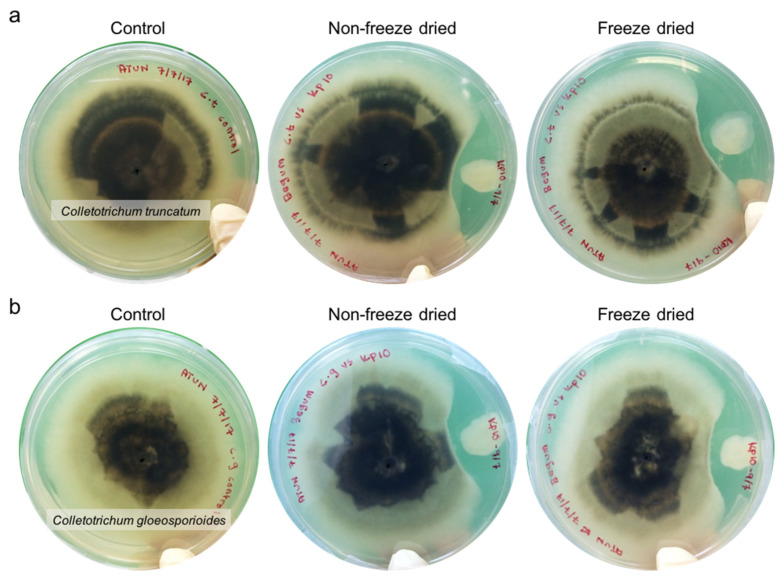
In vitro antifungal activity of *P. polymyxa* Kp10 against (**a**) *C. truncatum* and (**b**) *C. gloeosporioides.*

In this study, *P. polymyxa* Kp10 showed in vitro antifungal activity against the tested pathogens (Figure 3). As shown in Table 4, *P. polymyxa* Kp10 showed inhibitory effects in all the tested fungal pathogens with different efficacy. The dual cultures were incubated for five days and none of the fungal pathogens overlaid the bacterial colony. From the results, the highest percentage of inhibition of radial growth (PIRG) value (66.52%) was the freeze-dried *P. polymyxa* Kp10 against *Colletotrichum gloeosporioides,* while lowest PIRG value (60.18%) of freeze-dried *P. polymyxa* Kp10 against *C. truncatum* was recorded. The non-freeze-dried *P. polymyxa* Kp10 also demonstrated antifungal activity (Table 4) with PIRG values of 62.80% and 60.11% against *C. truncatum and C. gloeosporioides,* respectively. Likewise, Song et al. [38] reported that freeze-dried sulphate-reducing bacteria showed higher enzymatic activity in sulphate (SO_4_^−^) reduction compared to the fresh culture. Our findings were in accordance with several other studies on the antifungal activity performed by *Paenibacillus* species [8,39,40]. Jeong et al. [41] reported that *P. polymyxa* E681 produced an antibiotic known as polymyxin. Further, Karpunina et al. [42] proved that *P. polymyxa* 1460 has the ability to produce metabolites called lectins. Lectins are recognized to enhance cellulose degradation in plant cells and increase β-glucosidase activity in the wheat root cell wall. Moreover, most of the antimicrobial substances produced by *P. polymyxa* are peptides [40]. Meanwhile, *P. polymyxa* strains WR-2 and SQR-21 produced high β-1.3-glucanase when stimulated by the low concentration of uric acid produced by fungus *Fusarium oxysporum* [43]. Further, *Paenibacillus* strain producing a hydrolytic enzyme was also used for attacking a fungal cell wall containing β-1.3-glucan, chitin, and 11% protein [8].

## 3. Materials and Methods

### 3.1. Microorganism and Cell Growth Curve Preparation

The bacterium, *P. polymyxa* Kp10, used in this study was provided by Bioprocessing and Biomanufacturing Research Centre (BBRC), Universiti Putra Malaysia, Serdang. The strain was stored at −80 °C in 20% (*v*/*v*) glycerol as stock cultures. The strain was grown on an M17 agar plate and incubated for 48 h at 37 °C. A colony from the plate was picked and placed into 10 mL of M17 broth in a 15 mL centrifuge tube, then incubated at 37 °C for 18 h. The culture was used as inoculum culture for cell growth analysis. The culture was inoculated (5%) into M17 broth (100 mL) and incubated at 37 °C for 28 h at 150 rpm. Samples were collected starting at 6 h and sampled for every 2 h after until it stopped at 28 h. The number of cells is recorded as the average of the colony-forming unit (log CFU/mL) which is referred to as cell viability [22] and was used to plot the growth curve vs. the incubation time. The sampling was performed in triplicate.

### 3.2. Protective Agents Screening and Preparation of Protective Agents

Skim milk, lactose, sucrose, and Soytone were used as protective agents and distilled water was used as a control. In this study, all the protective agents were freshly prepared. The protective agents were autoclaved for 5 min at 121 °C. Skim milk (20% *w*/*v*), lactose (10% *w*/*v*), sucrose (30% *w*/*v*) and Soytone (15% *w*/*v*) were screened as the protective agents. The selection of the protective agents was based on the survival rate of the cell after freeze-drying in the preliminary experiments of this study (data shown in Table 1). The selected protective agents were identified by a central composite design (CCD). Table 5 displays all 20 different combinations of the selected protective agents for optimization.

### 3.3. Experimental Design for Optimization of Protective Agent Combination Using RSM

In this study, the considered cell viability of *P. polymyxa* Kp10 after freeze-drying was mainly affected by types and concentrations of the protective agent. To determine the optimal protective agent combination, the ranges of skim milk concentration (10%–30% *w*/*v*), lactose (5%–15% *w*/*v*), and sucrose (15%–40% *w*/*v*) were chosen based on the previous preliminary test and several other reported studies [11,28,30,40].

A five-level two-variable (Table 5) CCD with three replicates at the center point was conducted to determine the optimum combination of protective agents using RSM. Cell viability of *P. polymyxa* Kp10 was used as a response for the experiments. Analysis of variance (ANOVA) was used for regression analysis of variance. Table 6 depicts that each factor in the CCD was used at different levels (−1.1682, −1, 0, 1, 1.682). In performing the regression analysis and graphical experiments, Design Expert^®^ software version 7.0.0 (Stat-ease Inc, Minneapolis, MN, USA) was used.

### 3.4. Culture Preparation for Freeze-Drying

The strain was inoculated at 5% (*v*/*v*) in fresh M17 broth (500 mL) in a 1 L Erlenmeyer flask incubated at 37 °C for 22 h at 150 rpm. Cells were harvested by centrifugation within 22 h at 10,000× *g* for 15 min at 4 °C. The supernatants were discarded, and the cell pellets were washed once with sterilized 0.02 M phosphate buffer, pH 7.2. The cell pellets were centrifuged again before they were resuspended in different combinations of sterile protective agents. Then, 2 mL of bacterial suspension was placed in a 5 mL sterilized vial in triplicate and kept at −28 °C for 18 h. The frozen samples were desiccated in a pilot-scale freeze-dryer machine (Epsilon 1-8D, Martin Christ, Osterode am Harz, Germany) at 0.450 mbar vacuum, with a pre-freezing temperature of −40 °C and drying temperature of 20 °C for 48 h.

### 3.5. Recovery of Freeze-Dried Cells and Cell Viability Determination

The freeze-dried cell samples were reconstituted to their original pre-freeze-dried volumes by adding M17 broth and incubating at 37 °C for 1 h. For cell viability determination, serial decimal dilutions of each reconstituted sample (10^−1^ to 10^−6^ CFU/mL) in M17 broth were prepared and plated onto M17 agar plates, which were then incubated at 37 °C for 24 h. Colonies on the plate were counted and represented as colony-forming unit per volume (CFU/mL) [16]. Survival rate (%) after the freeze-drying process was calculated as follows [37]:

$$\mathrm{Survival}\;\mathrm{rate}\;\left(\%\right)=\frac{N_{o}-\left(N_{o}-N\right)}{N_{o}}\times 100\%$$

where N is the viable cell count after freeze-drying (FD) (CFU/mL); N_o_ is the viable cell count before FD (CFU/mL); FD represents freeze-drying.

### 3.6. Antifungal Activity

*P. polymyxa* Kp10 was screened for its in vitro antifungal activity against *C. gloeosporioides* and *C. truncatum* using a dual culture test based on the percentage of inhibition of radial growth (PIRG) [44]. A mycelia agar disc, 5 mm in length, from a 5-day-old culture was placed at the center of a Petri dish containing potato dextrose agar (PDA) medium. Plates were incubated at 27 °C for 48 h. A loopful of *P. polymyxa* Kp10 from 24 h of M17 agar plate culture was taken and streaked in a 3 cm circle from the mycelia agar disc on the same PDA plate and incubated for 5 days. *P. polymyxa* Kp10 in freeze-dried powder form was tested in the assay. Data for PIRG was recorded during the incubation period by measuring the radius of *C. gloeosporioides* and *C. truncatum* agar disc using the following formula:

$$Percentage\;of\;inhibition\;of\;radial\;growth\;\left(PIRG\right)=\frac{R1-R2}{R1}\times 100\%$$

where *R*1 indicates the radial growth of fungal agar disc in the control plate; *R*2 refers to the radial growth of the fungal agar disc in the dual culture plate [41].

## 4. Conclusions

In conclusion, the cell viability of *P. polymyxa* Kp10 after freeze-drying could be improved using an optimal concentration of protective agents. The optimal combination was found to be a mixture of lactose (10% *w*/*v*) and sucrose (27.5% *w*/*v*) that resulted in 5.839 log CFU/mL after freeze-drying. *P.polymyxa* Kp10 has proved to inhibit fungal phytopathogens through a dual culture test with the potential to be used as a biofungicide. Nevertheless, this study requires further works focusing on *P. polymyxa* Kp10 as a biofungicide in crop plantation to determine the stability of freeze-dried *P. polymyxa* Kp10 during field applications.

## Figures and Tables

**Table 1:** Effect of protective agents on cell viability of *P. polymyxa* Kp10 before and after freeze-drying.

Protective Agent	Viable Cell (log CFU/mL)		Survival Rate (%)
	* Before Freeze Drying	After Freeze Drying	
Skim milk (20% *w*/*v*)	8.050	7.185	89.26
Lactose (10% *w*/*v*)	8.000	7.022	87.78
Sucrose (30% *w*/*v*)	8.097	6.732	83.14
Soytone (15% *w*/*v*)	7.574	5.236	69.13
Distilled water (control)	7.468	4.648	62.24

* After the cell pellets mixed with the protective agents.

**Table 2:** A sequential model sum of squares.

	Sum of Squares	df	Mean Square	*F*-Value	*p-*Value (Prob > *F*)	
**Mean vs. Total**	661.95	1	661.95			
**Linear vs. Mean**	2.590 × 10^−4^	3	8.632 × 10^−5^	0.022	0.9954	
**2FI vs. Linear**	4.605 × 10^−3^	3	1.535 × 10^−3^	0.34	0.7960	
**Quadratic vs. 2FI**	0.058	3	0.019	505.45	<0.0001	Suggested
**Cubic vs. Quadratic**	1.940 × 10^−4^	4	4.851 × 10^−5^	1.54	0.3032	Aliased
**Residual**	1.892 × 10^−4^	6	3.154 × 10^−5^			
**Total**	662.02	20	33.10			

**Table 3:** Analysis of variance (ANOVA) and model coefficients.

Source	Sum of Squares	df	Mean Square	*F* value	*p*-Value (Prob > *F*)	
**Model**	0.063	9	6.998 × 10^−^^3^	182.58	<0.0001	Significant
**A-sucrose**	1.231 × 10^−^^4^	1	1.231 × 10^−^^4^	3.21	0.1034	
**B-skim milk**	9.761 × 10^−^^6^	1	9.761 × 10^−^^6^	0.25	0.6247	
**C-lactose**	1.261 × 10^−^^4^	1	1.261 × 10^−^^4^	3.29	0.0998	
**AB**	3.001 × 10^−^^4^	1	3.001 × 10^−^^4^	7.83	0.0189	
**AC**	4.005 × 10^−^^3^	1	4.005 × 10^−^^3^	104.49	<0.0001	
**BC**	3.001 × 10^−^^4^	1	3.001 × 10^−^^4^	7.83	0.0189	
**A^2^**	0.017	1	0.017	435.37	<0.0001	
**B^2^**	0.015	1	0.015	399.93	<0.0001	
**C^2^**	0.037	1	0.037	957.67	<0.0001	
**Residual**	3.833 × 10^−^^4^	10	3.833 × 10^−^^5^			
**Lack of Fit**	2.985 × 10^−^^4^	5	5.969 × 10^−^^5^	3.52	0.0968	Not significant
**Pure Error**	8.483 × 10^−^^5^	5	1.697 × 10^−^^5^			
**Cor Total**	0.063	19				

**Table 4:** Antifungal activity of non-freeze-dried and freeze-dried *P. polymyxa* Kp10 against fungal pathogens in dual culture after five days growth.

Fungal Pathogen	Non-Freeze-Dried		Freeze-dried	
	Inhibition Distance (mm)	Antagonism (PIRG)	Inhibition Distance (mm)	Antagonism (PIRG)
*Colletotrichum truncatum*	13.03	62.80%	13.95	60.18%
*Colletotrichum gloeosporioides*	14.8	60.11%	12.42	66.52%

Note: Radius of *C. truncatum* and *C. gloeosporioides* without *P. polymyxa* Kp10 (control) were 35.03 mm and 37.10 mm, respectively. PIRG: percentage of inhibition of radial growth.

**Table 5:** Protective agent combination based on a central composite design (CCD).

Run	Type	Factor 1 (X_1_)	Factor 2 (X_2_)	Factor 3 (X_3_)
		Sucrose	Skim Milk	Lactose
**1**	Center	0 (27.5%)	0 (20%)	0 (10%)
**2**	Center	0 (27. 5%)	0 (20%)	0 (10%)
**3**	Center	0 (27.5%)	0 (20%)	0 (10%)
**4**	Center	0 (27.5%)	0 (20%)	0 (10%)
**5**	Center	0 (27.5%)	0 (20%)	0 (10%)
**6**	Center	0 (27.5%)	0 (20%)	0 (10%)
**7**	Axial	0 (27.5%)	0 (20%)	1.682 (18%)
**8**	Axial	0 (27.5%)	0 (20%)	−1.682 (2%)
**9**	Axial	−1.682 (6.48%)	0 (20%)	0 (10%)
**10**	Axial	1.682 (48.52%)	0 (20%)	0 (10%)
**11**	Axial	0 (27.5%)	1.682 (37%)	0 (10%)
**12**	Fact	0 (27.5%)	−1.682 (3%)	0 (10%)
**13**	Fact	−1 (15%)	1 (30%)	−1 (5%)
**14**	Fact	1 (40%)	−1 (10%)	1 (15%)
**15**	Fact	−1 (15%)	−1 (10%)	−1 (5%)
**16**	Fact	1 (40%)	1 (30%)	1 (15%)
**17**	Fact	−1 (15%)	−1 (10%)	1 (15%)
**18**	Fact	−1 (15%)	1 (30%)	1 (15%)
**19**	Fact	1 (40%)	−1 (10%)	−1 (5%)
**20**	Fact	1 (40%)	1 (30%)	−1 (5%)

**Table 6:** Actual factor levels corresponding to coded factor levels for three variables.

Factor	Symbol	Actual Factor Level at Coded Factor of				
		−1.682	−1	0	1	1.682
**Skim milk (%)**	X_1_	3.18	10.00	20.00	30.00	36.82
**Lactose (%)**	X_2_	1.59	5.00	10.00	15.00	18.41
**Sucrose (%)**	X_3_	6.48	15.00	27.50	40.00	48.52
